# Microperimetric analysis of eyes after macular hole surgery with indocyanine green staining: a retrospective study

**DOI:** 10.1186/s12886-023-03161-3

**Published:** 2023-10-24

**Authors:** Sung Yeon Jun, Mingui Kong

**Affiliations:** 1 Department of Retinal service, Hangil Eye Hospital, Incheon, South Korea; 2 Department of Ophthalmology, Samsung Medical Center, Sungkyunkwan University School of Medicine, Seoul, South Korea; 3 Department of Ophthalmology, Kangbuk Samsung Hospital, Sungkyunkwan University School of Medicine, 29, Saemunan-ro, Jongno-gu, 03181Korea Seoul, South Korea

**Keywords:** Indocyanine green dye, Internal limiting membrane peeling, Macular hole, Microperimetry, Toxicity

## Abstract

**Background:**

Indocyanine green (ICG) aids in the visualization of the internal limiting membrane (ILM). Retinal damage from ICG dye toxicity has been reported through in vitro and in vivo studies. However, the clinical toxic effect of ICG during macular surgery has not been functionally evaluated. In this study, we evaluated functional and structural changes in retinal sensitivity and retinal thickness associated with ICG toxicity using microperimetry before and after ICG-assisted ILM peeling in patients with macular holes.

**Methods:**

ICG staining was performed only on the macular area below the horizontal line connecting the fovea and optic disc. ILM peeling was performed over the entire macular area inside the vascular arcade. Visual acuity assessment, spectral domain optical coherence tomography, and microperimetry were performed at baseline and one, three, and six months postoperatively. The mean retinal sensitivity of four macular areas was calculated and analyzed.

**Results:**

Eleven eyes were included. Macular holes were successfully closed in all patients. Six months postoperatively, retinal sensitivity improved insignificantly in Area 1 (ICG−/ILM−) and Area 2 (ICG−/ILM+) but decreased in Area 4 (ICG+/ILM−). Three months postoperatively, retinal sensitivity significantly decreased in Area 3 (ICG+/ILM+; 26.63 ± 1.80 vs. 25.52 ± 2.08 dB, p = 0.036). However, the statistical significance of this result was lost six months after the surgery (p = 0.059). The change of Gc-IPL thickness in Area 3 was significantly different compared to Area 2 at post-operative 3- and 6-months (p = 0.01, 0.05).

**Conclusions:**

Retinal sensitivity decreased three months after ICG-assisted ILM peeling. However, the statistical significance was lost six months after surgery. ICG staining can be performed with caution during macular hole surgery.

## Background

Internal limiting membrane (ILM) peeling improves anatomic closure rates and visual outcomes of macular hole surgery [1]. Indocyanine green (ICG) aids in the visualization of the ILM through selective staining, facilitating the removal of the membrane from the retina [2]. However, retinal damage from ICG dye toxicity has been reported through in vitro and in vivo studies. The mechanisms of toxicity are still unclear but have been postulated as follows: direct biochemical injury to the ganglion or neuroretinal cells [3], retinal pigment epithelium cells [4], and superficial retinal vessels [5]; apoptosis and gene expression alterations of either the retinal pigment epithelium cells or neuroretinal cells [6]; osmolar effect on the vitreoretinal interface [7]; light-induced injury [8]; and mechanical cleavage effect [9]. Furthermore, the clinical toxic effect of ICG during macular surgery has not been functionally evaluated.

Microperimetry comprises an automatic real-time tracking system for compensated eye movement and functionally evaluates the sublocation of fundus imaging [10]. It has already been shown to have good efficacy and provide detailed information on macular function, especially in patients with macular disorders [11]. Moreover, compared with best-corrected visual acuity (BCVA), retinal sensitivity has a reportedly more significant association with reading ability in patients with fundus disease [12, 13]. Therefore, this study aimed to evaluate the functional changes in the macular area associated with ICG toxicity using microperimetry to compare the retinal sensitivity of patients with macular holes before and after ICG-assisted ILM peeling.

## Methods

This study was approved by the Institutional Review Board (IRB) of Hangil Eye Hospital in Korea (IRB number: IRB-22,005) and adhered to the principles of the Declaration of Helsinki. This was a retrospective, randomized comparative interventional study, and all patients provided informed consent.

### Patient selection

#### Inclusion criteria

The study included patients diagnosed with a macular hole between May 2020 and December 2021 who underwent ILM peeling surgery with ICG staining.

### Exclusion criteria

Patients with pre-existing macular disease (myopic maculopathy and age-related macular degeneration) apart from the macular hole or with other ophthalmic diseases (uveitis, retinal vascular disease, optic neuropathy, and glaucoma), high refractive errors (i.e., high myopia [ < − 6 D] and hyperopia [ > + 3D]), previous history of ocular trauma and surgery, and significant media opacities that interfered with microperimetry were excluded.

### Surgical intervention

**Fig. 1:**
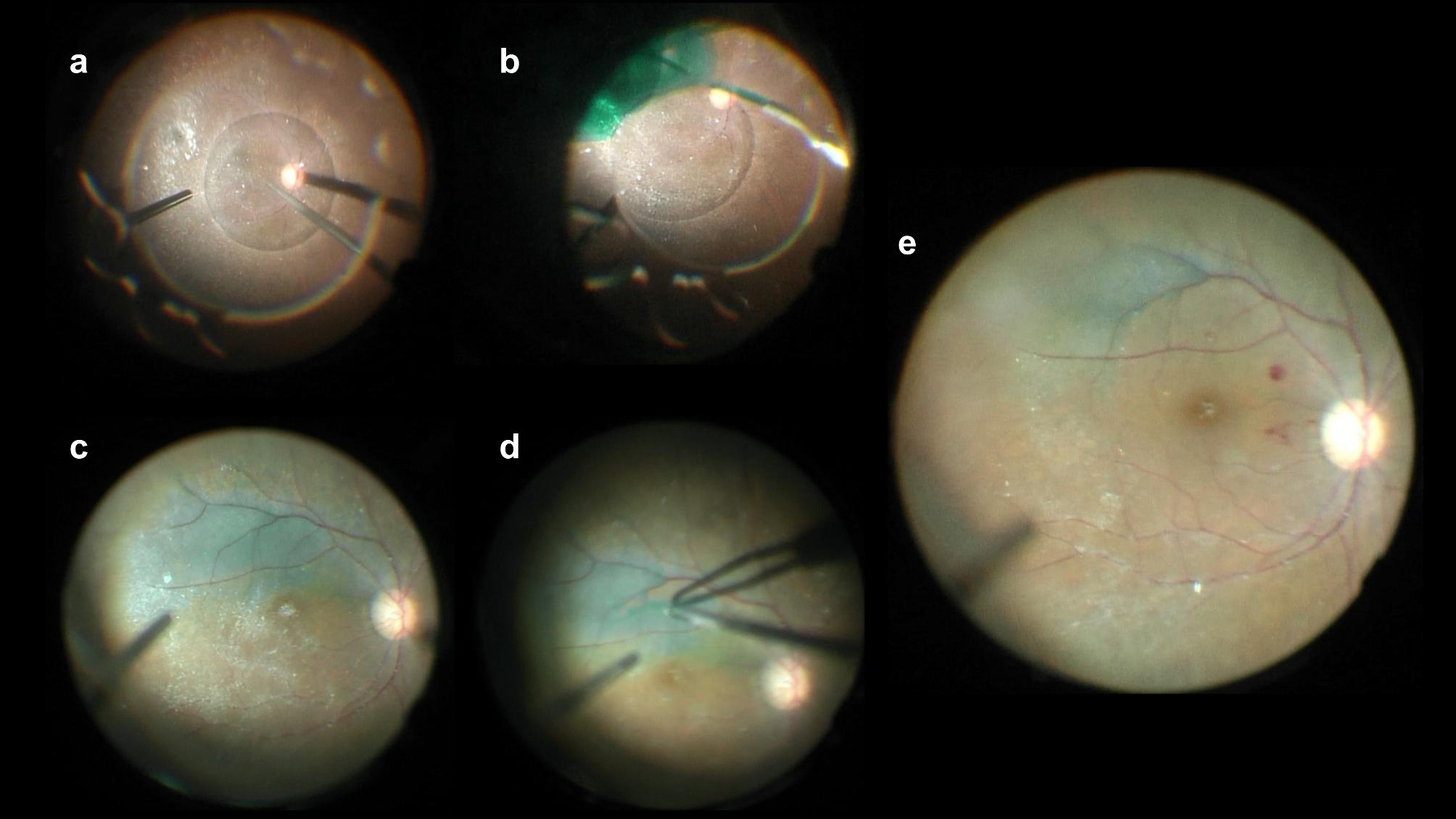
**Surgical method: a microscopic finding in the operating room** A microscopic finding in the operating room. The upper part of the photo represents the lower part of the patient’s eye. (**a**) Intravitreal perfluorocarbon liquid injection. (**b**) The eyeball is tilted, the superior portion is covered with perfluorocarbon liquid based on the horizontal line connecting the fovea and the optic disc, and indocyanine green (ICG) staining on the area below the horizontal line is performed. (**c**) The view after perfluorocarbon liquid removal; only the lower area is stained with ICG dye based on the horizontal line. (**d**) The internal limiting membrane (ILM) is peeled using forceps. (**e**) The ILM was peeled off from the entire macular area inside the vascular arcade

All surgeries were performed by one surgeon (MK). Based on the surgeon’s preference, all patients underwent cataract surgery to ensure a clear view for macular surgery. Phacoemulsification was performed before vitrectomy, and a monofocal intraocular lens was inserted. There was no eventful cataract surgical complication among patients. All patients underwent total vitrectomy with a 25-gauge, 3-port vitrectomy system (Alcon Labs, Fort Worth, Texas, USA). Trocar insertion was performed 3.0 mm from the limbus, and peripheral vitrectomy was performed following core vitrectomy. 0.25 mg/ml ICG dye was used to visualize the ILM before ILM peeling. perfluorocarbon liquid was injected first, followed by ICG dye injection. ICG staining was limited to the lower half of the macular area, based on the horizontal line passing through the fovea and the optic disc, with the help of perfluorocarbon liquid covering the upper half of the macular area without ICG staining. After washing out ICG and removing the perfluorocarbon liquid after 1 min of staining, ILM peeling was performed using conventional ILM peeling technique on the macular area, approximately 2disc diameter range in radius from macular hole, as seen in the image (Fig. 1). In all patients, low-dose 4% perfluoropropane gas (C_3_F_8;_ Teknomek, Istanbul, Turkey) was injected intravitreally and instructed for face down position after surgery. When macular hole size is not large and ILM peeling was performed using conventional method, low dose gas tamponade was preferred to quickly restore the patient’s vision.

### Ocular examination

All patients’ demographic data (age and sex) were recorded. Every patient underwent a complete ophthalmological examination before surgery, which included the following: BCVA in LogMAR, slit-lamp examination, intraocular pressure (IOP), dilated fundus examination, spectral-domain optical coherence tomography (OCT), and microperimetry. Postoperatively, a complete ophthalmological examination was performed on the first day and then in week 1, followed by monthly examinations. BCVA measurement, OCT, and microperimetry were conducted three months after vitrectomy.

### Microperimetry

**Fig. 2:**
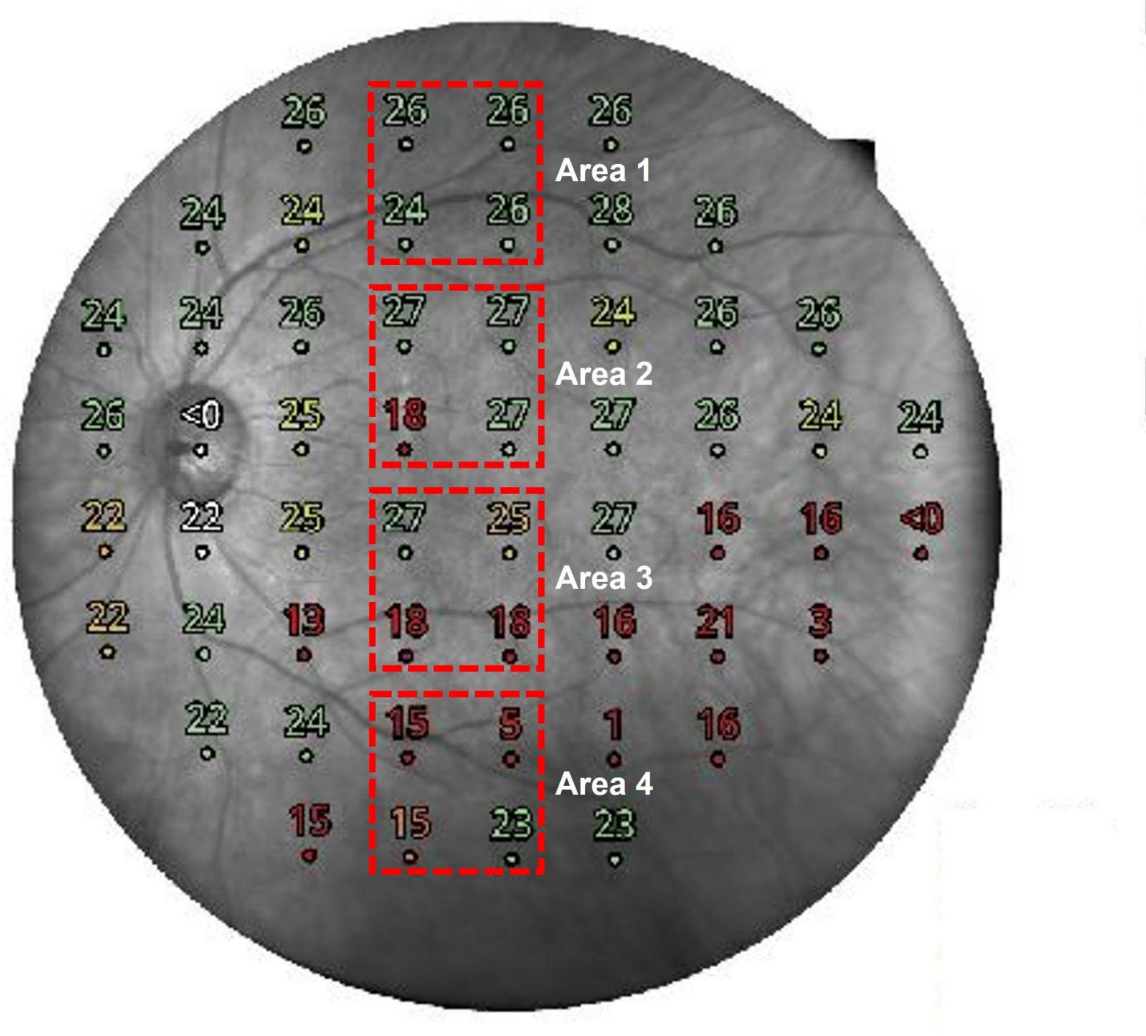
**Area classification and analysis according to the surgical method using compass microperimetry** The macular area was divided into four areas. Area 1 was around the superior arcade, where neither internal limiting membrane (ILM) peeling nor indocyanine green (ICG) staining was performed. Area 2 was inferior to Area 1 and above the fovea, where ILM peeling but not ICG staining was performed. Area 3 was below the fovea, where both ILM peeling and ICG staining were performed. Area 4 was around the inferior arcade below Area 3, where ICG staining but not ILM peeling was performed. The mean retinal sensitivity of four points within each area was manually calculated

Compass microperimetry (CMP; centerVue, Padova, Italy) was conducted in a dark room after pupil dilation with one drop of 1% tropicamide and 2.5% phenylephrine. It was performed with a double control for fixation. CMP evaluated 24 − 2 grids (54 locations spaced by 6 degrees), and the testing strategy was an adaptation of the Zippy estimation by sequential testing. CMP was considered reliable if the false-positive frequency was < 18%. Details of the microperimetric analysis are shown in Fig. 2. The foveal retinal sensitivity was obtained as the retinal sensitivity at one point in the fovea. Mean retinal sensitivity of Area 1, 2, 3 and 4 was measured as the average value of four points retinal sensitivity within the area. Area 3 was selected as four points within 2-disc diameter in the downward direction from macular hole, and area 2 was selected as four points within 2-disc diameter in the upward direction from macular hole. Area 4 was selected as four points outside 2-disc diameter in the downward direction from macular hole, and area 2 was selected as four points outside 2-disc diameter in the upward direction from macular hole. A superimposed fundus image automatically generated by CMP was used to confirm the ILM-peeled area (Fig. 2).

### Retinal layer thickness

Retinal layer thickness was measured using Spectralis-domain OCT, which automatically detect the fovea based on the ETDRS grid. The inbuilt Spectralis software, Heidelberg Eye Explorer automatically segments and measures the thickness of each retinal layer. Mean retinal layer thickness of total retina, inner retina, outer retina, retinal nerve fiber (RNFL), ganglion cell-inner plexiform (Gc-IPL), retinal pigment epithelium (RPE) was measured within ETDRS grid. Of the ETDRS grid, only the superior and inferior sections with a diameter of 3 to 6 mm was used for retinal thickness analysis, and each section corresponded to area 2 and 3 respectively in analysis.

### Statistical analysis

SPSS Statistics 23 (IBM Corp., Armonk, NY, USA) was used to conduct statistical analysis. The data are expressed as mean ± standard deviation. The comparison of average retinal sensitivity between the four areas was conducted using the Mann–Whitney U test. The Wilcoxon signed-rank test was used to examine the changes in values of each area from the baseline assessment to the follow-up assessment at three months. Statistical significance was set at a p-value less than 0.05.

## Results

### Baseline characteristics

Patient demographics are shown in Table 1. A total of 11 eyes from 11 patients were included. The mean age was 63.0 ± 7.6 years, and four patients were men (36.4%). All the eyes were phakic, and the lens opacity was below nuclear sclerotic grade 2 and cortical opacity grade 2. The mean BCVA was 0.38 ± 0.28 logarithm of the minimum angle of resolution (logMAR), and the mean IOP was 14.90 ± 2.38 mmHg. The average macular hole size was 220.12 ± 122.88 (range, 101–485) µm. The preoperative mean retinal sensitivity (MRS) of Areas 1, 2, 3, and 4 were 24.72 ± 2.52, 27.18 ± 1.10, 26.06 ± 2.13, and 22.59 ± 2.11 dB, respectively.

**Table 1:** Baseline characteristics of patients with macular holes

Baseline characteristics	Value
Number of patients	11
Age (years)	63.0 ± 7.6
Sex (male/female), n/n	4/7
Corrected visual acuity (logMAR)	0.38 ± 0.28
Refractive error (SE)	−0.01 ± 1.30
IOP (mmHg)	14.90 ± 2.38
Lens status (phakia), n/n	11/11
Macular hole size (µm , range)	220.12 ± 122.88(132, 395)

Data are expressed as mean ± standard deviation values unless otherwise specified. logMAR, logarithm of the minimum angle of resolution; SE, spherical equivalent; IOP, intraocular pressure

### Surgical outcomes of macular hole and cataract surgery

Compared with the preoperative examination results, patients showed improvement in BCVA six months after surgery (baseline vs. follow-up: 0.38 ± 0.28 vs. 0.16 ± 0.17 logMAR; p = 0.024). Foveal retinal sensitivity measured by microperimetry also significantly improved at 6 months after surgery (baseline vs. follow-up: 18.33 ± 14.42 vs. 28.11 ± 1.37; p = 0.012). The IOP was not significantly different between preoperative and postoperative examination at six months (baseline vs. follow-up: 14.90 ± 2.38 vs. 14.63 ± 2.73 mmHg; p = 0.905). Signs of successful macular hole closure were observed in all patients postoperatively, and there were no complications associated with pars plana vitrectomy, such as retinal breaks or detachment, glaucoma, and endophthalmitis.

### Analysis of retinal sensitivity in areas with regards to ICG staining and ILM peeling

Table 2 shows the longitudinal changes in retinal sensitivity in Areas 1, 2, 3, and 4. MRS values improved six months postoperatively compared with the baseline in Area 1 (ICG staining−/ ILM peeling−) and Area 2 (ICG staining−/ ILM peeling+), but the difference was not statistically significant (baseline vs. follow-up: Area 1, 24.47 ± 3.63 vs. 25.40 ± 4.09 dB; p = 0.262 and Area 2, 27.13 ± 1.67 vs. 28.04 ± 1.12 dB; p = 0.057). The MRS at Area 4 (ICG staining+/ ILM peeling−) decreased after surgery, but there was no statistical significance (21.77 ± 3.88 vs. 21.00 ± 4.32 dB, p = 0.812). However, in Area 3, where both ICG staining and ILM peeling were performed, the MRS value significantly decreased three months after surgery compared with baseline (baseline vs. follow-up: 26.63 ± 1.80 vs. 25.52 ± 2.08 dB, p = 0.036). However, the statistical significance was lost at six months postoperatively (baseline vs. follow-up: 26.63 ± 1.80 vs. 25.52 ± 1.95 dB, p = 0.059).

**Table 2:** Functional outcomes of macular hole surgery per the longitudinal changes in retinal sensitivity in Areas 1, 2, 3, and 4 according to the surgical methods

Variable	Treated eye
MRS (dB), Area 1 (ICG staining−/ILM peeling−)	
Baseline	24.47 ± 3.63
1 month	24.72 ± 2.52
3 months	24.63 ± 3.27
6 months	25.40 ± 4.09
p value^a^ (baseline vs. 1 month)	0.929
p value^a^ (baseline vs. 3 months)	0.918
p value^a^ (baseline vs. 6 months)	0.262
MRS (dB), Area 2 (ICG staining−/ILM peeling +)	
Baseline	27.13 ± 1.67
1 month	27.18 ± 1.10
3 months	27.20 ± 1.49
6 months	28.04 ± 1.12
p-value^a^ (baseline vs. 1 month)	0.943
p-value^a^ (baseline vs. 3 months)	0.812
p-value^a^ (baseline vs. 6 months)	0.057
MRS (dB), Area 3 (ICG staining+/ILM peeling+)	
Baseline	26.63 ± 1.80
1 month	26.06 ± 2.13
3 months	25.52 ± 2.08
6 months	25.52 ± 1.95
p-value^a^ (baseline vs. 1 month)	0.199
p-value^a^ (baseline vs. 3 months)	0.036*
p-value^a^ (baseline vs. 6 months)	0.059
MRS (dB), Area 4 (ICG staining+/ILM peeling−)	
Baseline	21.77 ± 3.88
1 month	22.59 ± 2.11
3 months	21.02 ± 3.72
6 months	21.00 ± 4.32
p-value^a^ (baseline vs. 1 month)	0.332
p-value^a^ (baseline vs. 3 months)	0.683
p-value^a^ (baseline vs. 6 months)	0.812

Data are expressed as mean ± standard deviation values unless otherwise specified. *: significant p-values. ^a^Comparison between the baseline and follow-up visit for each value (Wilcoxon signed-rank test). MRS, mean retinal sensitivity; ICG, indocyanine green; ILM, internal limiting membrane

### Analysis of retinal layer thickness changes in area 2 and 3

**Fig. 3:**
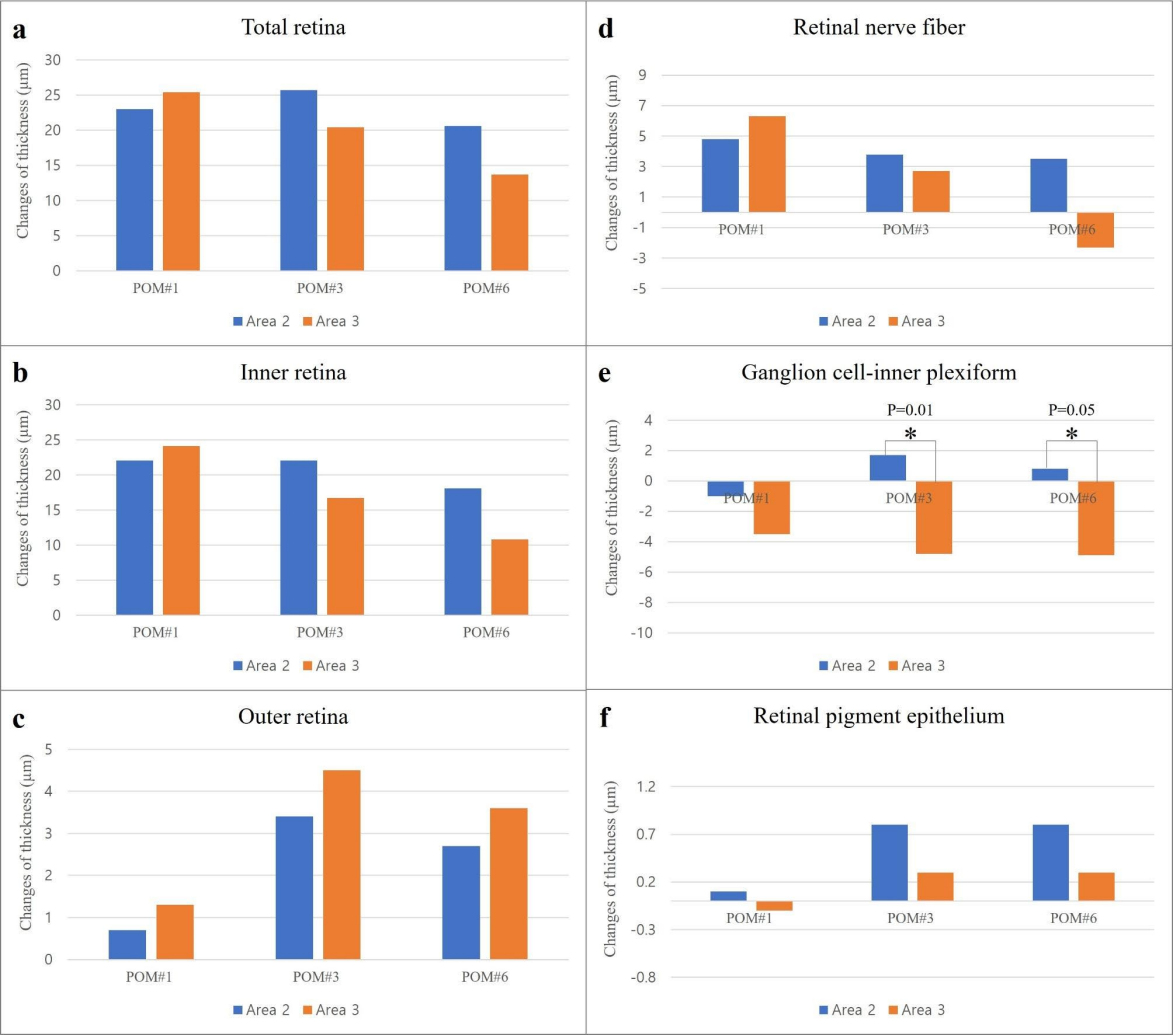
**The change of retinal layer thickness in Area 2 and 3 during follow-up period** The retinal layer thickness was measured using optic coherence tomography. The (**a**) total, (**b**) inner and (**c**) outer retinal thickness tended to increase after surgery, but there was no significant difference between Area 2 and 3 at all follow-up time points. (**d**) RNFL thickness tended to increase in both Area 2 and 3 at post-operative 1 and 3 months but decreased at post-operative 6-months only in Area 3. However, the change of RNFL thickness was not significantly different between Area 2 and 3. (**e**) Gc-IPL thickness in Area 3 decreased at post-operative 1-, 3- and 6-months. The change of Gc-IPL thickness in Area 3 was significantly different compared to Area 2 at post-operative 3- and 6-months (Area 2 vs. 3: post-operative 3 months, p = 0.01; post-operative 6 months, p = 0.05). (f) There was no significant difference in changes of RPE layer thickness between Area 2 and 3 at every follow-up time points

The changes of retinal layer thickness in Area 2 and 3 during the follow-up period are shown in Fig. 3. The total, inner and outer retinal thickness tended to increase after surgery compared to baseline, but there was no significant difference between Area 2 and 3 at all follow-up time points (Area 2 vs. 3: post-operative 1, 3 and 6 months, all p > 0.05). Gc-IPL thickness in Area 3 decreased at post-operative 1-, 3- and 6-months. The change of Gc-IPL thickness in Area 3 was significantly different compared to Area 2 at post-operative 3- and 6-months (Area 2 vs. 3: post-operative 1 month, -1.00 ± 5.96 vs. -3.50 ± 5.31, p = 0.34; post-operative 3 months, 1.70 ± 6.81 vs. -4.80 ± 4.58, p = 0.01; post-operative 6 months, 0.80 ± 6.74 vs. -4.90 ± 5.21, p = 0.05). RNFL thickness tended to increase in both Area 2 and 3 at post-operative 1 and 3 months but decreased at post-operative 6 months only in Area 3. However, there was no significant statistical difference in changes of RNFL thickness between Area 2 and 3 for 6 months follow-up period (Area 2 vs. 3: post-operative 1 month, 4.80 ± 5.51 vs. 6.30 ± 7.40, p = 0.63; post-operative 3 months, 3.80 ± 6.90 vs. 2.70 ± 7.80, p = 0.79; post-operative 6 months, 3.50 ± 6.41 vs. -2.30 ± 6.14, p = 0.06). There was no significant difference in changes of RPE layer thickness between Area 2 and 3 at all follow-up time points (Area 2 vs. 3: post-operative 1 month, 0.10 ± 1.28 vs. -0.10 ± 0.99, p = 0.68; post-operative 3 months, 0.80 ± 1.82 vs. 0.30 ± 0.67, p = 0.28; post-operative 6 months, 0.80 ± 0.91 vs. 0.30 ± 0.67, p = 0.16).

## Discussion

The present study reported the effects of ILM peeling and ICG staining on the retinal sensitivity of patients with macular holes after combined vitrectomy surgery. In patients, a significant decrease in retinal sensitivity was observed three months postoperatively in the retinal area where both ILM peeling and ICG staining were performed. However, the statistical significance compared with baseline values was lost six months after surgery.

Previous microperimetry studies have reported that in patients with macular holes, the MRS of the central 2-degree area improved three months after vitrectomy and ILM peeling surgery (p = 0.004) [14]. Moreover, the area around the macular hole where ILM peeling was performed reportedly had significantly improved MRS four months after vitrectomy and ILM peeling surgery compared with baseline (baseline vs. follow-up: 23.46 ± 3.01 dB vs. 27.14 ± 2.45 dB, p < 0.01) [15]. However, previous studies have only reported on the effect of ILM peeling. The present study is the first to report on the impact of ILM peeling and ICG staining on retinal sensitivity in patients with a macular hole.

The results of the present study revealed that only the area where both ICG staining and ILM peeling were performed showed a statistically significant decrease in retinal sensitivity at three months with a tendency to recover six months after surgery. And Gc-IPL thickness decreased significantly in area where both ICG staining and ILM peeling was performed at three and six months after surgery. Uemura et al. reported peripheral visual field defects after ICG-assisted ILM peeling [16]. ICG exhibited dose-dependent toxicity to retinal ganglion cells [3]. Residual ICG after ILM peeling may induce synergistic toxicity on Gc-IPL thinning and retinal sensitivity. Although it was not statistically significant, a decreasing in retinal sensitivity compared to baseline was observed 1 month after surgery. It is possible that retinal sensitivity decreased sub acutely over time due to a synergistic effect caused by ICG staining and ILM peeling. Also, considering that some retinal sensitivity improved with cataract surgery, retinal sensitivity may also have decreased 1 month after surgery. The effects on retinal sensitivity seemed to lose statistical significance over time. However, because the number of subjects in this study was small and there was still a significant difference in degree of margination at p = 0.059 at 6 months after the surgery, it is necessary to evaluate the longer-term changes in retinal sensitivity to determine the reversibility of toxicity after peeling.

Retinal sensitivity decreased in Area 4, where only ICG staining was performed, but it was statistically insignificant. A possible hypothesis is that the two procedures, ICG staining and ILM peeling, may have a synergistic retinal toxicity, rather than staining or peeling alone causing toxicity. In addition, the cataract surgery may have resulted in positive improvement of retinal sensitivity, partially deduct the decreased retinal sensitivity in area 3 and 4, resulting in no statistically significant difference in area 4.

In Areas 1 and 2, which were not stained with ICG, ILM peeling had no significant effect on retinal sensitivity. Although not statistically significant, retinal sensitivity of both areas improved after cataract combined vitrectomy surgery, regardless of ILM peeling. According to Qi et al., ILM peeling without ICG staining in patients with a macular hole did not cause retinal desensitization [15]. Some studies have reported decreased retinal sensitivity and microscotomas after active ILM peeling due to possible mechanical trauma with the forceps [17]. However, in the present study, we did not observe reduced retinal sensitivity after ILM peeling alone.

The present study had several limitations. First, this was a retrospective study. Second, cataract surgery may be a confounding factor. However, all patients had a mild lens opacity of N2C2 or below and cataract surgery was performed due to the surgeon’s preference to secure macular surgery view. The decrease in retinal sensitivity is in the opposite direction to the expected improvement from cataract surgery. So, the decrease of MRS still can be considered as a meaningful change. Third, this study had a small sample size. Therefore, the statistical power may not have been sufficient to appreciate the difference, and further studies with larger sample sizes are required. However, because the changes in the functional outcomes following the use of different surgical techniques were analyzed for each area in one patient not among other patients, it was possible to compensate for this limitation. We are planning a study comparing ICG dye with brilliant blue G under long term follow up in future.

## Conclusions

In summary, even in the retinal tissue of the same patient, retinal sensitivity decreased after three months in the area where both ILM peeling and ICG staining were performed. However, since the statistical significance was lost six months after the surgery, we conclude that ICG staining can be performed, with caution, during macular hole surgery if deemed essential by the surgeon.

## Data Availability

The datasets used and/or analysed during the current study are available from the corresponding author on reasonable request.

## Abbreviations

ILM: Internal limiting membrane
ICG: Indocyanine green
IOP: intraocular pressure
BCVA: best-corrected visual acuity
IRB: Institutional Review Board
CMP: Compass microperimetry
logMAR: logarithm of the minimum angle of resolution
MRS: mean retinal sensitivity
